# Microglial regional heterogeneity and its role in the brain

**DOI:** 10.1038/s41380-019-0609-8

**Published:** 2019-11-26

**Authors:** Yun-Long Tan, Yi Yuan, Li Tian

**Affiliations:** 1 Psychiatry Research Centre, Beijing Huilongguan Hospital, Peking University Health Science Center, Beijing, China; 2 Children’s Hospital of Capital Institute of Pediatrics, Beijing, China; 3 Institute of Biomedicine and Translational Medicine, Department of Physiology, Faculty of Medicine, University of Tartu, Tartu, Estonia

**Keywords:** Neuroscience, Psychiatric disorders

## Abstract

Microglia have been recently shown to manifest a very interesting phenotypical heterogeneity across different regions in the mammalian central nervous system (CNS). However, the underlying mechanism and functional meaning of this phenomenon are currently unclear. Baseline diversities of adult microglia in their cell number, cellular and subcellular structures, molecular signature as well as relevant functions have been discovered. But recent transcriptomic studies using bulk RNAseq and single-cell RNAseq have produced conflicting results on region-specific signatures of microglia. It is highly speculative whether such spatial heterogeneity contributes to varying sensitivities of individual microglia to the same physiological and pathological signals in different CNS regions, and hence underlie their functional relevance for CNS disease development. This review aims to thoroughly summarize up-to-date knowledge on this specific topic and provide some insights on the potential underlying mechanisms, starting from microgliogenesis. Understanding regional heterogeneity of microglia in the context of their diverse neighboring neurons and other glia may provide an important clue for future development of innovative therapies for neuropsychiatric disorders.

## Introduction

Constitution of different types of neurons in different nuclei or areas of the central nervous system (CNS), such as the cerebral cortical layers I–VI, is a well-known neurobiological phenomenon and has been enlightened by recently advanced high cell-resolution technologies [1, 2]. Glial cells, categorized into lineages of astrocytes, oligodendrocytes, and microglia, etc., collectively support neuronal health and viability. Whether or not glia derived from different brain regions are phenotypically and functionally distinct is comparatively less clear, but an increasing body of evidence suggests so. Following an earlier study that reported distinct expression patterns of brain genes by integrating the mouse Allen Brain Atlas with published cell type-specific transcriptome profiling data [3], several single-cell RNA sequencing (scRNAseq) studies have confirmed the spatial patterns of astrocyte- and oligodendrocyte-specific genes and have discovered molecularly distinct glial cells comprising both known major cell types and novel classes [1–5]. Multiple studies on astrocytes have further provided solid evidence on their region-selective roles in controlling physiological and pathological brain functions (reviewed in [6–9]). Moreover, regional changes in astrocyte- and oligodendrocyte-specific genes were found to predict aging of the human brain more precisely than neuron-specific genes [10].

**Fig. 1:**
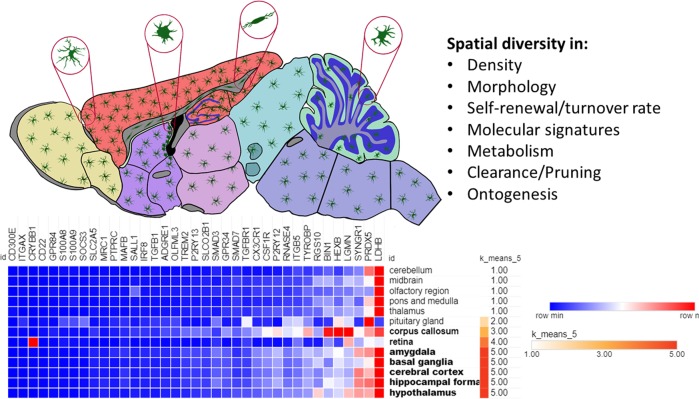
Regional heterogeneity of microglia in the brain. Microglia differ in their cell number, cellular and subcellular structures, molecular signature as well as relevant functions in different mouse brain areas. Microgliome genes were selected based on several seminal transcriptomic studies [25, 26, 63, 68, 107, 115, 140] and their RNAseq data in 13 mouse brain regions were retrieved from the human protein atlas. An expression heatmap was drawn in Morpheus (Broad Institute). Brain regions were clustered according to K-means of expression levels (grouping score at 5, Pearson’s method). The data are also provided in Supplementary Table 1

As a unique type of glia in the CNS, microglia are now appreciated as an important gardener and defendant of the CNS [11]. Although the past years witnessed rapid progression in microglial research, microglia have largely been regarded as a holistic entity or homogenously polarized population in both CNS developmental and diseased conditions so far. Nevertheless, microglial researchers, including us, have begun to investigate various aspects of microglial heterogeneities [12–19]. In awake of such increasing attentions on this phenomenon, this review focuses on recent advances in examining the diversity of microglial phenotypes across different compartments of the adult brain as well as the potentially causative intrinsic and extrinsic factors. Why do neuropsychiatrists need to know the distinct inter- and intra-regional features of microglia? We believe this helps for a better comprehension of psychiatric disorders and for developing more precise treatment strategies accordingly. To more clearly define the term “regional heterogeneity” that is our focus here (Fig. 1), and to discern it with other important aspects of microglial heterogeneities under various contexts, which are beyond our scope, we first briefly summarize the current scenarios in the following section.

## What does microglial regional heterogeneity concern?

Microglia are considered the most versatile inhabitants in the mammalian CNS with a multitude of broad functions, dynamically changing at cellular, subcellular and molecular levels to adapt to their ever-changing surroundings. Accompanied with such dynamic changes, complex and heterogeneous features of microglial functions in both physiological and pathological conditions also emerge. Microglial heterogeneity has multiple meanings, including temporospatial [11–14, 20, 21] and gender-specific [22, 23] differences in regards to cellular origin [11, 24], colonization [18, 20, 21], abundancy [11, 20], morphology along with mobility and motility [11, 18, 20], as well as gene expression [16, 17], which ultimately are translated into diversified physiological and pathological functions [15, 19]. Due to the space limit here, we will not elaborate on these respective topics but direct readers to the cited review articles above, with a hope to set ours as a standpoint, from which CNS structural and functional maintenance done by microglia, across temporal stages and between genders, can be extrapolated on psychiatric disorders, and neurological diseases as well.

Nevertheless, we would like to notify the readers that even the term “regional heterogeneity” itself may have several layers of meanings. As detailed described in the following sections, microglia may differ among and within specific brain regions; they may further consist of the same subtype of cells but located in different anatomical regions with i.e., different abundancies; or alternatively, of different subtypes in different anatomical regions. Microglia that functionally differ from each other are suggested to mix even within the close vicinity of the same anatomical site [12, 15, 19], an interesting phenomenon that still needs experimental proof on the reason. Noteworthy, with the advent of scRNAseq and mass cytometry, traditional classifications of brain cell types and subtypes, including not only neurons but also glia, have been turned over [1–5], thereby making fundamental paradigm shift on brain taxonomy. In the case of microglia, a broad range from one to eight different clusters or subtypes in the adult mouse brains have been reported by scRNAseq [1, 2, 25–29], with their spatial features not all clearly depicted yet. Contradictory findings on microglial regional heterogeneity in molecular signatures also exist (see section “Heterogeneity in microglial molecular signature across CNS regions”).

Furthermore, the underlying mechanisms of microglial regional heterogeneity—alongside a clear interpretation on its potential significance for normal CNS functions and for disease development—remain elusive. For instance, whereas astrocytic development has been convincingly shown to depend on not only specified neuronal cues in different brain regions, but also distinct radial glia lineages that retain embryonic positional information into adulthood [8], whether this is also true for microglia awaits demonstration. Overall, the above-described complications may make appropriate interpretations of research findings challenging. Hence, in the next sections, we thoroughly summarize the current knowledge on various aspects of microglial regional heterogeneity and aim to provide some deeper and provocative perspectives on its contributing factors, as well as its potential role for maintaining or restoring brain functions in normal and diseased conditions.

## Heterogeneity in microglial density across CNS regions

Studies by immunohistochemists have already observed differences in microglial densities in brain compartments in early 1990s (Table 1). An initial investigation using F4/80 in mice found higher density in the hippocampus, olfactory bulb, basal ganglia and substantia nigra (SNr); lower in the fiber tracts, cerebellum and brainstem; and average in the cerebral cortex, thalamus and hypothalamus [30]. Using an antibody against lipocortin 1 (LC1), another earlier study reported the density was higher in the forebrain, lower in the midbrain, and lowest in the brainstem and cerebellum [31]. However, an analysis of OX-42-positive rat microglia reported the highest density in the mesencephalic SNr as opposed to the hippocampus and cortex [32]. Recent work using ionized calcium-binding adapter molecule-1 (Iba1) confirmed a higher microglial density in the frontal cortex and a lower one in the cerebellum [33, 34]. Using flow cytometry, we have also observed a clear regional difference in microglial numbers, the cerebellum and spinal cord (SC) having much lower abundance than the cortical regions (Supplementary Fig. 1). Besides, studies using voluntary wheel-running [35] or chronic restraint [36] or unpredictable [37] stress to treat rodents reported changes in microglial density in selective brain regions. Likewise, human microglia also demonstrate regional heterogeneity in both early embryonic and adult brains, featuring less CD68- and major histocompatibility complex (MHC) II-positive cells in the cerebellum compared with regions such as the mesencephalon [38] and medulla oblongata [39], as well as lower density in cerebral gray matter (GM) compared with white matter (WM) [39].

**Table 1:** Basal regional features of microglia in the healthy rodent and human brains

	Frontal brain: cortex, striatum, NAc	Hippocampus	SVZ, CVO	Midbrain: thalamus, hypothalamus, VTA, SNr	Hindbrain: cerebellum, brainstem	SC
Density	High-average [30, 31, 33, 34]; Lower in human cerebral GM than WM [39]	High [30, 31]; Lower in CA3 than CA1 [21]	High in CVOs [49]	Average [30, 31] or high [32]; Higher in SNr than VTA [65]	Low [30, 31, 33, 34]; Higher in cerebellar nuclei and granular layers [30, 40]; Less CD68+ & MHCII+ in human cerebellum [38, 39]	Low [136, 137]
Morphology (ramification)	High [33, 34]	High [33, 34]	Amoeboid [30, 49, 52]	Higher in SNr than VTA [65]	Low [33, 34]	Average; Cell smaller in dorsal horn [138]
Molecular expression CX3CR1	High (Fig. 1)	High (Fig. 1)	Unknown	Low-median (Fig. 1)	Low (Fig. 1)	Unknown
TREM-2	High [58]	High [58];	Low [58]	Low (Fig. 1)	Low (Fig. 1)	Unknown
Phagocytic or immune activating genes (*Trem 3* etc.)	Low [28, 65, 68]	Low-median [28, 68]	High [49, 52, 67]	High in VTA [65]	High [28, 68]	Unknown
Immune inhibitory genes (*Sirpa etc.), Cd206*, *P2ry12*	High [68]; Higher in human GM [67];	Median [68]	High IL-4 [49, 52]; Low P2ry12 [58]	Low *P2ry12* (Fig. 1)	Low *P2ry12* (Fig. 1)	Low Sirpa [136]
Others (NF-κB, CD11b, MHCII, *Tim3*, etc.)	Higher in human WM [66, 75, 76]	Median [59, 62, 63, 136]	High [49]	Median [59, 62, 63, 136]	Median [59, 62, 63, 136]	High [59, 62, 63, 136]
Cellular functions: Proliferation/replenish after ablation	Both fast [42]	Both fast [46, 48]	Both fast [44, 45, 104]	Replenish fast [42]	Fast [48] /replenish slower [42]	Replenish fast [42]
Protrusion toward ATP/Phagocytosis/pruning	Fast protrusion [52]/Low lysosome content in NAc [65]	High surveillance [74, 82]	Slow protrusion [52]	High lysosome content in SNr [65]/High pruning in thalamus [117]	Less surveillance [34]/High clearance [77]	High pruning [139]
Ontogenesis	Hoxb8^±^ [101]; Sensitive to IL-34 [111, 112] but not CSF1 [113]	Sensitive to IL-34 [111, 112];	Unknown	Hoxb8^−^, [101]; Insensitive to IL-34 [111, 112]	Hoxb8^−^, [101]; Sensitive to CSF1 [113] but not IL-34 [111, 112]	Unknown

Heterogeneity in microglial density was found to exist even in the same region, such as in different histological layers within the cerebellum, as detected by either F4/80- or NDPase-positivity [30, 40]; the density was higher in the cerebellar nuclei than in the cortex [40], and the molecular layer less densely populated than the granular layer and WM in the cerebellar cortex [30, 40]. Likewise, a later paper also found that Iba1-positive microglial density differed within the mouse hippocampus, with lower density in the CA3 region than in the CA1 region and dentate gyrus; conversely, no interregional differences were detectable in the dorsal hippocampus [41]. Microglial ablation studies using genetic or pharmacological approach have also revealed that repopulation of microglia in different CNS regions diversify after ablation. For example, a diphtheria toxin receptor (DTR)-mediated genetic approach rapidly ablated microglia in all studied areas including the cortex, cerebellum and SC within 3 days, but residual microglia recovered more rapidly in the cortex and SC than the cerebellum within 14 days [42].

It is still unclear why the CNS needs such variations of microglial density in different regions. Noteworthy, the density of microglia in different brain regions is in line with the overall glia-to-neuron ratio across the brain. The number of nonneuronal cells has a linear relationship with the brain structure mass across mammalian species. In humans, the glia-to-neuron ratio in the cerebral cortex is 3.72 (72% glia), while that in the cerebellum is only 0.23 (19% glia), implicating tightly coordinated migration and proliferation of glial types [43]. It is equally speculative whether  microglial variability stems from the different rates of cell proliferation and/or cell death of mature residential microglia, or from the (re)population of different microglial progenitor cells derived from different embryonic brain regions (see section “What may contribute to regional heterogeneity of microglia:nature versus nurture?”). Earlier studies showed that microglia isolated from the neurogenic subependymal zone and hippocampus proliferated massively in vitro, whereas microglia isolated from nonneurogenic brain regions did not and that both intrinsic and extrinsic factors may contribute to the proliferation [44, 45]. A recent study on both the mouse and human brains observed that microglial self-renewal was maintained by coupled proliferation and apoptosis with about 0.5–2.5% of proliferation rate, and that the rate varied across regions in the young adult mouse brain, with microglia in the dentate gyrus having the highest [46]. Another recent study detecting the level of ^14^C in genomic DNA of microglia isolated from the human brain showed that 28% of human microglia, with an average life-span of 4.2 years, were renewed every year and most of which continued to do so indefinitely [47]. Using a multicolor fluorescent mapping system by crossing CX3CR1^creER^ mice with R26^Confetti^ reporter mice, Tay et al. observed that microglia established a dense network with highly variable turnover rates in multiple brain regions, which challenges the concept of microglial longevity in a healthy brain [48]. They reported subtly (one-to-two cycles) more active cell division in hippocampal and cerebellar microglia compared with cortical microglia, and further found that a random self-renewal shifted toward a selective clonal microglial expansion during neurodegeneration induced by facial nerve axotomy [48].

## Heterogeneity in microglial morphology across CNS regions

Microglia undergo constant morphological remodeling to engage with other CNS elements for synaptic pruning and clearance of tissue debris under both physiological and pathological circumstances. Classification based on morphological changes using both fixed and real-time imaging techniques has been a key approach for evaluation of microglial function. Microglia are usually morphometrically graded as ramified (numerous thin processes, radial branching), primed (thickened processes, increased polarity and proliferation with reduced secondary branching), reactive (thickened stout processes with highly reduced branching), or amoeboid (rounded soma with no branching) based on standard morphological criteria [11].

An early study in 1990 already discovered a more than five-fold variation in the density of microglial processes between different regions [30] (Fig. 1, Table 1). Microglial morphologies, although normally ramified with extended branches in most brain regions, vary significantly, even within the same anatomical entity [30, 33, 34, 40, 49]. For instance, microglia in the cerebellum contained relatively smaller soma and bigger cytoplasm area but lower ramification complexity [33, 34] and covered area [33] than those in the striatum, hippocampus and frontal cortex. In addition, microglia showed different sizes and ramification patterns both within and between different histological layers of the cerebellar cortex [40]. Studies have also found that, unlike those in the other brain regions, microglia in brain regions with an incomplete blood-brain barrier (BBB) [50], such as the median eminence [51], circumventricular organs (CVOs) [30, 49] and subventricular zone (SVZ) [52], displayed an amoeboid form with fewer or shorter branched processes under normal conditions in adult mice.

A previous interesting study isolated microglia from frozen tissue sections of the corpus callosum of neonate and juvenile rat brains by laser capture microdissection. Comparison between transcriptomes of amoeboid and ramified microglia found that equivalent levels of cytokines and chemokines were produced by both subtypes, but only amoeboid microglia uniquely expressed genes involved in cell cycle and migration, implying that morphologically polarized subtypes of healthy microglia may not differ in their immune properties but may confer different synaptic functions [53]. Consistently, using a time-lapse in vivo two-photon imaging, a recent study suggested that differences between cerebral and cerebellar microglial distribution and morphology may underlie decreased surveillance of Purkinje neurons by cerebellar microglia [34]. Likewise, cortical microglia were found to extend their processes much faster toward the site of adenosine tri-phosphate (ATP) injection than microglia in the SVZ and rostral migratory stream, which were less branched and expressed immune effectors characteristic of an alternatively activated phenotype with a simultaneously lower level of purinergic receptor P2RY12 [52].

Communication between neurons and microglia is crucial for optimal regulation of behavior and physiology. In this regard, fractalkine (CX3CL1)—secreted from neurons and its microglial target—CX3CR1 receptor, which represent an important neuron-microglia regulatory pathway for brain structure and function [54], may underly the regional heterogeneity of microglial morphology. *Cx3cr1* gene is relatively enriched in the cortical regions, basal ganglia and hypothalamus as compared with noncortical regions (Fig. 1, Supplementary Table 1), whereas CX3CL1 is highly expressed in the cortical layers and basal ganglia too [55]. Supportively, a recent study on morphological assessment of microglia in CX3CR1-deficient mice showed region-selective changes, primarily within the hypothalamus and cortex as compared with wild type (WT) animals [56]. However, a different study found no correlation between CX3CR1-deficiency and microglial density, distribution, morphology or motility in either adolescent or young adult mice across brain regions [57].

## Heterogeneity in microglial molecular signature across CNS regions

Regional heterogeneity of microglial gene signatures has been the most thoroughly characterized microglial feature so far (Fig. 1, Table 1). Region-specific expression of key microglial receptors at their basal levels was already discovered by microglial researchers in early 2000s. For instance, fractalkine was found to be almost absent in the hindbrain but constitutively expressed at high levels in the forebrain, particularly in the hippocampus, basal ganglia, olfactory bulb and layers II, III, V, and VI of the cortex [55]. Likewise, triggering receptor expressed on myeloid cells-2 (TREM-2) was found to be expressed variably both between and within healthy brain regions, with the lateral entorhinal and cingulate cortices showing the highest TREM-2 positivity [58]. Another study detected that microglia expressed the markers CD11b, CD45, CD86, and CCR9 significantly more in the SC than the hippocampus [59], and a later study showed that *Cd68* mRNA expression was higher in the olfactory bulb than in the hippocampus and amygdala [60].

By contrast, BBB-less regions had the lowest percentages of TREM-2-positive microglia [58], whereas microglia in the CVOs strongly and constitutively expressed M1 markers CD16/32 and CD86 as well as M2 markers CD206 and Ym1 [49]. The findings also demonstrated that compared with cortical microglia, microglia in the SVZ supported neurogenesis and neural migration and were more alternatively activated, i.e., M2-like, by expressing higher levels of anti-inflammatory cytokines interleukin (IL)-4 and -10 [52]. Fundamental differences in microglial development and microglia-mediated neuroinflammation between the brain and SC have also been noticed [61]. Microglia in the SC were found to express higher levels of immune molecules than microglia in the brain both at steady state [62, 63] and upon viral infection [62]. We also found that microglial expression of CD11b/c and MHCII were constitutively higher in the SC than the cortical regions in both naïve rats [63] and mice (Supplementary Fig. 1). Moreover, region-specific molecular change of aging-induced microglial priming was further highlighted in one study, showing that aged mice had greater upregulation of microglial activation markers CD11b, CD68, CD11c, F4/80, and FcyRI in the WM compared with the GM [64].

With the advent of high-throughput cell sorting and RNAseq technologies, several recent works on transcriptomic profiling of microglia from both the mouse and human brains have further demonstrated the existence of regional differences in microglial gene expression profiles [65–68] (Fig. 1, Supplementary Table 1). Among them, a pivotal study showed that microglia had distinct region-dependent transcriptional identities that changed in a region-dependent manner during aging [68]. Furthermore, differences in bioenergetic and immunoregulatory pathways, such as *Siglec5*, *Cd33*, and *Sirpa* (CD172a) were downregulated, while some other genes were upregulated in cerebellar microglia compared with striatal and cortical microglia in the young adult brain. By contrast, in the aged brain, an augmentation of a distinct cerebellar phenotype with a concomitant loss in distinction of hippocampal immunophenotype was found [68]. A recent study on mouse basal ganglia also reported the two above-cited microglial molecular pathways and highlighted that transcriptomes in the cortex, SNr and nucleus accumbens (NAc) differed from that in the ventral tegmental area (VTA), particularly with genes involved in mitochondrial and oxidative functions, as well as Fcγ-receptor-mediated phagocytosis and phagosome maturation. Furthermore, several other microglial features also differed within the basal ganglia nuclei and such region-specific phenotypes occurred as early as during the second postnatal week [65].

Region-specific microglial gene expression in the human brain was also reported in a previous study and glia-specific genes were found to predict aging more precisely than neuron-specific genes [10]. Moreover, differential gene expression profiles between the WM and GM in both humans and mice have also been described (see section “Heterogeneity in microglial functions across CNS regions: from phenotypes to actions”). It is still unclear, however, whether the variations in expressions of microglial molecules are similar at mRNA and protein levels in the brain. A recent study on the human brains revealed that protein-RNA relationships varied, with generally increased magnitudes of difference in abundancy of proteins among brain regions compared with that of RNAs; furthermore, structurally similar cortical regions were different in the abundances of receptor-associated and resident plasma membrane proteins that were not readily observed in RNA expression data [69]. Noticeably, a very recent human study using mass cytometry discovered that the SVZ and thalamus accommodated a more activated phenotype of microglia by expressing higher levels of dozens of microglial markers than the cerebellum as well as the temporal and frontal cortical lobes, and further identified CD11c, CD206, CD45, CD64, CD68, CX3CR1, HLA-DR, and IRF8 as key markers for the detection of human microglia regional heterogeneity [67].

However, the phenomenon of regional differences in molecular signatures may be equivocal, as recent scRNAseq work has also created some inconsistency regarding populational diversity of microglia under steady state of the mouse brain. For instance, Matcovitch-Natan et al. found a striking level of microglial homogeneity during brain development [63]. A lately study instead reported that early postnatal microglia were more heterogeneous; by contrast, most adult mouse microglia expressing homeostatic genes were remarkably similar in transcriptomes, regardless of brain regions [25]. Another scRNAseq analysis of adult mouse cortical cells neither found phenotypical heterogeneity of microglia [2]. In their scRNAseq analysis, Masuda et al. lately found that compared with juvenile microglia, adult microglial clusters showed a more homogenous distribution across regions, but a minor cluster of CST3^−^SPARC^−^IBA1^+^ microglia was more prevalent in the adult cerebellum and corpus callosum compared with the cortex and hippocampus [28]. Likewise, an analysis of hippocampal microglia isolated from an Alzheimer’s disease mouse model did not detect heterogeneity of microglia in control littermates but did identified heterogenous responses of microglial populations to neurodegeneration [70]. Whether discrepant results on adult microglial heterogeneity between bulk and scRNAseq studies is due to technological issues, e.g., limitations in cell acquisition number and sequencing depth, as well as isolation artifact, awaits to be addressed in the future.

## Heterogeneity in microglial functions across CNS regions: from phenotypes to actions

Despite the various acknowledged heterogenous features of microglia as described above, a direct link between phenotypes and functions is not always straightforward, as for instance amoeboid and ramified microglia were found to produce equivalent levels of cytokines and chemokines [53] in neonate mice and ramified dystrophic (fragmented, senescent) microglia were associated with aging and neurodegeneration in humans, rather than activated amoeboid microglia [71]. Nevertheless, some morpho-molecular phenotypes are associated with microglial functions in both brain development and diseased processes (Table 1). For example, at postnatal (P) day 10, transformation from amoeboid to ramified microglia was accompanied by the loss of transcription factor Runx1 [72]; at P28, microglia are fully ramified with concomitant decrease in microglial density and transcriptomic maturation that retains into adulthood in mice [63]. In maturing fetal human microglia, an increase in ramification accompanied with gradual loss of several CD markers was also observed [73].

However, direct evidence on the consequences or functionalities of region-specific features of microglia is still insufficient and a global topography on different functionalities in association with heterogenous microglial phenotypes is currently lacking. Encouragingly, evidences from multiple studies jointly indicate that for instance microglia in the cortical GM and WM are phenotypically and functionally different. As microglia are the primary phagocytes of the CNS, their actions of tissue debris clearance and pruning of neuronal circuits during development and throughout adulthood provide a key mechanism for CNS structural and functional plasticity, and have been a major focus in studies of various brain diseases [74]. In this regard, microglia were previously found to express high levels of MHCII [75] and Tim-3 (a cell surface protein that regulates macrophage activation and promotes immunological tolerance) [76] in the corpus callosum compared with the cerebral cortex in humans. One recent scRNAseq human study found higher levels of type-I interferon genes in the GM but higher levels of NF-κB pathway genes in the WM; transcriptional changes in microglia isolated from multiple sclerosis patients also differed between the GM and WM [66]. Furthermore, a unique cluster of axonal tract- or oligodendrocyte-associated microglia, expressing *Spp1*, *Gpnmb*, *Igf1*, *Cd68*, etc. and with an amoeboid morphology, was found to be specifically enriched in the corpus callosum of neonatal mice by two recent studies [25, 26]. These suggest a specific regulation of myelin uptake by WM microglial subtype, which may have significant clinical implications (see section “How may regional heterogeneity be associated with psychiatric disorders?”).

A recent epigenetic study on mouse microglia reported that cerebellar, but not striatal or cortical, microglia exhibited a high level of basal clearance activity associated with an elevated degree of cerebellar neuronal death [77]. Several other transcriptomic profiling works have also depicted regional differences in microglial expression of phagocytosis-related genes [65, 68]. These jointly imply that regulation of neurotransmission and synaptic functions by microglia could be region-specific.

A glimpse of such site-dependency of microglial synaptic functions was very recently provided by a study of the retina, where microglia were found to primarily reside in two distinct synaptic compartments, namely the outer and inner plexiform layers, of the retinal neural parenchyma [78]. The study also showed that only microglia located in the inner plexiform layers contributed to the normal processing of cone-driven visual information as detected by electroretinography. However, as no changes in the number of synapses between the retinal cone and bipolar cells were detected, the findings implied a possible qualitative defect in these synapses [78]. It is, for example, suggested that failed clearance together with exaggerated release of glutamate by activated glial cells jointly lead to aberrant extra-synaptic signaling through ionotropic and metabotropic glutamate receptors, which ultimately result in synaptic dysfunction, and loss; furthermore, glutamate diffusion outside the synapse can lead to the loss of synaptic fidelity and specificity of neurotransmission [79]. Microglia may also sense and control the turnover of other key neuromodulators released by synapses, such as ATP and ADP, and, via balancing their ratios through activation of purinergic receptors and adenosine enzymes, modulate neuronal excitability [80].

Microglia may, therefore, affect functional neurotransmission without changing synaptic numbers, and hence contribute to circuit dysfunction and behavioral pathology. This has been demonstrated in the microglial receptor CX3CR1 [81], as CX3CR1-deficiency only decreased synaptic pruning transiently [82]. Studies also showed that CX_3_CR1 rescued microglial phagocytosis [83], as well as impairments in hippocampal long-term potentiation [83] and short-term memory induced by stress [84]. Interestingly, CX3CL1 was reported to be differentially expressed across different mouse brain regions [55, 85], suggesting that CX3CL1-CX3CR1-mediated synaptic modulation may be region-specific and associated with selective alterations in brain circuits at different developmental stages.

Besides phagocytic and pruning functions, it should be mentioned that microglia are also regionally pivotal for a multitude of other important brain functions, such as neurogenesis in the SVZ, guided neuronal migration to the olfactory bulb, and cerebral angiogenesis [11, 18, 20]. Indeed, it is the function that eventually matters, such that there was even a suggestion that microglial identity should be based on functional classifications in future research [19]. However, based on currently circumstantial and limited knowledge on microglial functions and the punctual focus of a certain research project, this strategy may practically turn out to be challenging. An outstanding yet unaddressed question is whether microglial phenotypical and functional features are interchangeable within the same brain region and/or across different regions, under either normal or pathological condition. As introduced in this and previous sections, regional heterogeneity can be very complicated and dynamic, with current comprehension of it still being obscure. But plasticity and transformation are possible and may provide an important mechanism for disease onset and development. A relevant hint on this notion is that many brain cytokines secreted by astrocytes and microglia, such as interleukin (IL)-1β and tumor necrosis factor (TNF) α, are involved in neuroinflammation only in pathological conditions, but rather regulate brain structural and functional homeostasis, such as long-term potentiation and synaptic scaling normally [86].

So far, studies revealing the heterogenous functions of microglia and microglial molecules in different human brain regions have been rare. In an imaging genetics study, respective roles of TNFα receptors, i.e., TNFR1 and TNFR2, in regulation of hippocampal and striatal morphologies in healthy human subjects was explored [87]. TNFα is one of the key microglia-derived cytokines that are known to regulate synaptic transmission and cognition [88]. TNFR1 and TNFR2 are microglial receptors exerting opposite effects on neuronal survival, with TNFR1 being neurodegenerative and TNFR2 neuroprotective [89]. Interestingly, *TNFRSF1A* (encoding TNFR1) single nucleotide polymorphisms (SNPs) rs4149576 and rs4149577 were found to have highly significant genotypic associations with striatal but not hippocampal volume, whereas *TNFRSF1B* (encoding TNFR2) SNP rs1061624 yielded a significant association with hippocampal but not striatal volume [87]. In addition, a most recent transcriptomic study evaluating cell-type-specific contribution to cortical thickness in the adolescent human brain showed that inter-regional profiles in cortical thickness were 70% dependent on those in the expression of genes marking CA1 pyramidal cells, astrocytes, and microglia; and these genes were also related to age-dependent cortical thinning [90].

## Different origins and routes of entrance and migration of microglial precursors in the CNS?

**Fig. 2:**
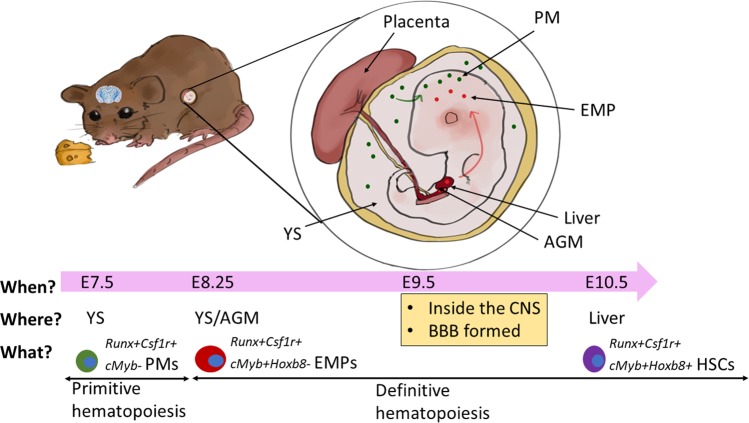
Possible different regional ontogenesis and entry routes of microglia in the developing mouse brain. Microglia, along with other tissue macrophages, are generated in successive waves of myelogenesis during mouse embryonic (E) development. The first wave of microglia is generated by *Runx*- and *Csf1r*-dependent, *cMyb*-independent primitive macrophages (PMs) in the yolk sac (YS) at E7.5, whilst the second wave is generated by *cMyb*-dependent erythromyeloid progenitors (EMPs) at E8.25 from the YS as well as the aorta–gonad–mesonephros (AGM) and fetal liver at E8.25-E10, during which definitive hematopoiesis happens. Adult microglial (or microglia-like) cells may also come from *cMyb*-dependent fetal liver monocytes in mouse [141] and embryonic hematopoietic stem cells (HSCs) in zebrafish [94]. A transient appearance of fetal liver-derived microglia in the neonate mouse brain was also found [46]. Microglial precursors from the primitive and definitive waves migrate into the brain at E9.5 and self-sustain throughout adulthood [91, 92]. It is possible that microglia deriving from different origins and in different waves may enter and occupy different niches in the developing brain. For example, Hoxb8^−^ and Hoxb^+^ microglia came from primitive and definitive hematopoiesis, respectively, and infiltrated the brain at different E stages in different brain areas [101]

Recent studies on microglial development have revealed diversified microglial origins and invasion routes into the embryonic brain, as well as factors regulating their differentiation and homeostasis upon brain invasion. However, many developmental aspects of microglia still lack a clear depiction and we hereby tentatively summarize the current knowledge generated by other researchers, which may explain or shed light on the mechanisms underlying regional diversity of adult microglia (Fig. 2, Table 1).

The question of microglial origin has witnessed a series of turns of conclusions in the past 30 decades and is still somewhat confusing up to date, changing from hematopoietic myeloid origin shared by monocyte-derived tissue macrophages to yolk sac (YS)-derived *c-Myb*-dependent erythromyeloid progenitors (EMPs) at mouse embryonic stage (E8.25–10) [11, 24]. Lately, mouse microglia were found to also arise from YS-derived *c-Myb*-independent primitive macrophages (PM) at an earlier stage (E7–8.25), before definitive hematopoiesis starts [91, 92]; at E9.5, they infiltrate and persist in the embryonic brain, before the BBB is formed, and self-sustain throughout adulthood [91, 92] (Fig. 2). Studies on zebrafish have observed a parallel pattern of microgliogenesis with mouse [93, 94]. Microglial progenitor cells enter the mouse cortex in several waves during E10.5–E17.5 [18, 95], a stage when bone marrow-derived *c-Myb*-dependent hematopoietic stem cells (HSCs) materialize in mice. Studies on the entrance routes of microglia suggest that they might infiltrate from “hot spots” such as the meninges, cerebral ventricles and blood vessels in mice [18, 21]. In this regard, whether macrophages, or their subtypes, which are located in the meninges, SVZ/ventricles and perivascular space and known to be morphologically and molecularly very different from parenchymal microglia [19, 27], would become microglial progenitors or at least carry some of such features in adulthood circumstances is an interesting notion to take.

Post-mortem studies on the developing human brains have also suggested a step-wise entry and colonization of microglia during the early gestational weeks (GW), preceding the appearance of the brain vasculature and the BBB as well as neurogenesis in various brain areas [96, 97]. In particular, earlier studies showed several entry routes of human amoeboid microglia-macrophages into the brain rudiment at an early or late embryonic stage, i.e., from the leptomeninges [98, 99], the ventricular lumen [98] and the choroid plexus [97] at early stage (GW4.5–5.5), and from the parenchymal vasculature in the cerebellum and diencephalon at GW5.5 [99] but in the telencephalon only at late stage (GW12–13) [98]. Amoeboid microglia-macrophages entering from the ventral routes were restricted to the WM (intermediate zone), then migrated and matured both radially and tangentially toward the immature subplate layer and cortical plate not until GW22, whereas pial cells populated the prospective layer I cortex [73, 97]. They were also found to arise earlier in the mesencephalon than other brain areas at GW8 [38]. Finally, amoeboid microglia-macrophages were found to colonize in the SC from the meninges at GW9, later than in the cerebrum [73]; and during GW18–24, gray matter microglia were ramified while WM microglia were amoeboid in the SC [100].

To make the story of microglial origin more complicated, recent studies pointed out the possibility of non-YS origin of microglia during early postnatal development. For instance, an earlier report found distinct origins for embryonic and adult microglia in zebrafish, with embryonic microglia arising from the rostral blood island (a primitive hematopoiesis organ similar as the YS) [93]. Instead, adult zebrafish microglia arose from the ventral walls of the dorsal aorta where the second or definitive wave of hematopoiesis originates in zebrafish and mouse [93]. A recent work on zebrafish uncovered PM as the unique source of embryonic microglia, which however were replaced by definitive microglia originating from HSCs later and persisted throughout adulthood [94].

A study on mice also found that fetal liver-derived monocytes infiltrated the brain at the peak of P3 and transiently contributed to the resident microglial pool, but were rapidly depleted at P6, hence not contributing to the adult microglial population [46]. Furthermore, a recent study on *Hoxb8* in microglia found that unlike canonical Hoxb8-negative microglia, Hoxb8-positive microglial progenitors were generated during the second wave of primitive hematopoiesis in the YS at E8.5–10, then expanded in the aorto-gonad-mesonephros and fetal liver, where the definitive hematopoiesis occurs, and infiltrated the brain not until E12.5, which was much later than canonical microglia. The authors further demonstrated that Hoxb8-positive hematopoietic progenitor cells taken from the fetal liver were competent to give rise to microglia in vivo [101]. *Hoxb8* mutant mice were earlier detected of compulsive grooming and hair removal (trichotillomania) resembling the obsessive-compulsive disorder (OCD) in humans, and this distinct behavior was attributed to *Hoxb8* mutant microglia [102], which were recently found to cause corticostriatal circuit defects in mutant mice [103].

Is the issue of developmental origin of microglia important for understanding regional heterogeneity of microglia in adulthood? The answer would be yes as microglial precursors of different origins and in different waves may use different routes to infiltrate into and migrate in the early developmental brain (Fig. 2). A previous work on mice found that most Iba1+ microglial progenitors were located within the meninges at E10.5 and clustered into the VZ/SVZ where chemokine CXCL12 was highly expressed at E14.5–18.5 [104]. Furthermore, inactivation of microglial CXCR4 by its specific antagonist at E10.5 led to a decrease in microglia in the SVZ/VZ at E18.5, indicating a key role of CXCL12-CXCR4 axis in the distribution of microglia in specific regions of the embryonic brain [104]. More interestingly, the above-cited *Hoxb8* study found that Hoxb8-positive microglia were more abundant in the cortex than in other brain regions during embryogenesis and early postnatal development, and although both Hoxb8-negative and -positive populations were very similar molecularly, they showed distinct brain distributions, with Hoxb8-positive microglia more abundant in the dorsal striatum and the olfactory bulb than other regions such as the frontal cortex, ventral pallidum and substantia nigra [101]. It is hence possible that microglia derived ontogenically from distinct progenitor lineages may enter and populate different brain areas at different embryonic stages that sustain into adulthood. However, this speculation still needs more experimental supports.

## What may contribute to regional heterogeneity of microglia: nature versus nurture?

**Fig. 3:**
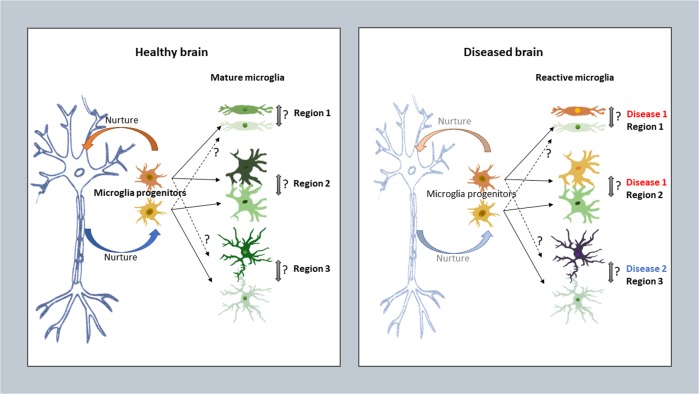
Can regional heterogeneity of microglia be plastic and dynamic under healthy and diseased conditions? In the healthy adult CNS, a balanced neuron-microglia interaction may be necessary, or unnecessary, for microglia derived from progenitors of singular or multiple lineages to maturate and maintain their individual identities in different regions. Whether their phenotypes and the related functions are interchangeable under normal condition is unclear. Under pathological conditions, microglia at different locations, or even in the same place, probably do not all react homogenously toward different stimuli at different places and may individually change features during the process of the disease development. But whether they can dynamically transform into another subtype within the same location or even into a subtype that may carry different anatomical features is also uncertain

When and how does regional heterogeneity of microglia start to happen? Solid answers to these questions are not accessible yet. Differences among astrocytes that allow them to fulfill specific brain functions have been suggested to be both autonomous and attributable to neurons that actively determine the features of astrocytes via sonic hedgehog, etc.-mediated mechanisms [7, 8]. It is intuitive that regional heterogeneity of microglia is also a likely result of their residential environment, particularly of interaction with neurons or neural progenitor cells, but intrinsic mechanisms are also possible (Figs. 2 and 3). It is widely known that when microglia are cultured in vitro, their morphology, gene signature and functions are dramatically altered, revealing ultra-sensitivity of microglia to environmental changes [105–107]. Two recent studies on mouse and human microglia, respectively, further revealed environment-dependent epigenetic landscapes specifying programs of microglial gene expression [77, 107]. Another recent work on mice found that region-specific phenotypes can be re-established following genetic or pharmacological ablation and repopulation of microglia in the adult basal ganglia, indicating that local cues play an ongoing role in shaping microglial diversity [65]. In another recent study, a dynamic yet discrete self-renewal of mature microglia in the healthy mouse CNS was unraveled, and the importance of local neurodegenerative cues in guiding a rapid expansion of only selected microglial clones was highlighted [48]. Yet, it is still intriguing what kind of cues in a pathological milieu these could be and how they could selectively regulate microglial proliferation.

Multiple neuron-microglia crosstalk mechanisms may underpin the regional features of microglia [108]. Selective inhibition of colony-stimulating factor 1 receptor (CSF1R) or targeted genetic deletion of CSF1R resulted in elimination of 90–99% of all microglia brain-wide, demonstrating that microglial proliferation and survival in the adult brain are physiologically dependent on CSF1R signaling [104, 106, 109, 110]. Two simultaneous earlier studies reported that IL-34, an alternate ligand of CSF1R which was expressed mainly by neurons in the brain, seemed to be vital in maintaining microglial numbers in a regional manner, as microglial density was reduced in IL-34-deficient mice only in the cortex and striatum, but not in the cerebellum and brainstem [111, 112]. A very recent study in CSF1-deficient mice also confirmed that CSF1 depletion affected the number of microglia in the cerebellum but not the frontal cerebral cortex [113]. It would be important to further understand whether this region-specific dependency on CSF1 and IL-34 is caused by difference in the neuronal expression of IL-34, or its microglial receptor CSF1R, or both, among brain areas. Although CX3CL1-CX3CR1 signaling has been implicated as another key neuron-microglia communication axis during synaptic plasticity [54], it is still debatable whether this axis provides a universal or region-specific mechanism and whether its critical function is limited only to certain developmental stages. Previous studies showed miscellaneous functions of CX3CR1 in this regard, and, as described above, studies on the role of CX3CR1 in regulation of region-selective changes of microglial morphology yield controversial results [56, 57].

Noteworthy, besides neurons, astrocytes may represent another major cause for regional diversity of microglia, as they carry this distinct nature themselves. But evidence supporting this notion is currently still limiting. Three recent studies have nevertheless highlighted the utmost importance of TGF-β, a cytokine that is mainly produced by astrocytes in the brain [114], for regulating activation [106, 110] and differentiation [26, 115] of microglia in mice. Moreover, the IL-1 family cytokine produced by developing astrocytes, IL-33, was shown to modulate microglial gene expression and promote microglial synaptic pruning during development [116]. Guiding cues that drive the differences of microglia between the BBB-intact and BBB-less brain regions as well as between the brain and the SC may also come from the peripheral circulation [61]. Collectively, the above data suggest that heterogeneity of microglia likely arises from differences in the connecting neurons, neighboring glia and stem cells, as well as the infiltrated blood-derived molecules through fenestrated capillaries in certain BBB-less regions, such as the CVOs.

Besides extrinsic factors, it is also intuitive that cell-autonomous mechanisms may drive the differentiation of microglia into different subtypes, as peripheral bone marrow-derived blood cells do. But could there be any intrinsic factors? Evidence to support this postulation is still limited (see section “Different origins and routes of entrance and migration of microglial precursors in the CNS?”). Interestingly, a very recent scRNAseq study depicting nine major microglial subpopulations found that unlike other metabolically active and proliferative microglial subpopulations, a microglial subpopulation was persistently associated with unmyelinated axon tract during early brain development [26], implying the existence of intrinsic program in region-selectivity of microglia. Besides, the most recent demonstration on Hoxb8-positive (about 40% of total microglia) and -negative microglia that were nonredundant in their developmental origin, brain-invasion pattern and function also provides a relevant evidence [101].

## How may regional heterogeneity be associated with psychiatric disorders?

CNS diseases often occur with a disease-specific spatial pattern, the origin of which is obscure, however. As has been reviewed above, regional heterogeneity of microglia exists in various facets of microglial phenotypes and functions. Considering the importance of microglia for the CNS, the phenomenon of its regional heterogeneity raises concerns as whether they uniformly or selectively influence CNS functions and contribute to CNS diseases (Fig. 3). It is highly expected that baseline regional differences of microglia among the broad compartments of the CNS, or even within the small area of a focal nuclei, may finely tune the neuronal/glial functions and neural circuitry, hence contributing to the onset, development and treatment response of respective CNS diseases. Regarding the significance of this on various psychiatric disorders, limited knowledge is available currently. We nevertheless hereby gather relevant in vitro and in vivo laboratory evidences on animal models and patients to discuss on this topic.

Several previous studies have demonstrated that microglia in different brain regions responded differently to psychiatry-related aging [64, 68, 117] and stress [36, 37]. For the development of stress-associated affective disorders, regional diversity of microglia may be directly involved, as has been demonstrated in several animal models. For example, loss of progranulin was found to trigger preferential elimination of inhibitory synapses by microglia only specifically in the ventral thalamus and hence caused OCD-like behavior in mice [117]. Similar OCD-like phenotype has also been described for *Hoxb8* mutant mice, which showed a striking region-specific distribution of microglial subtypes (see section “Different origins and routes of entrance and migration of microglial precursors in the CNS?”). In addition, CX3CR1-deficient mice showed reduced anxiety-like behaviors, as well as increased fear acquisition and reinstatement relevant to post-traumatic stress disorder as compared with WT mice [56]. Furthermore, compared with WTs, CX3CR1-deficient mice were resistant to stress [83, 84, 118], implicating an intriguing detrimental role of CX3CR1 in eliciting susceptibility to psychiatric disorders. We found that region-specific transformation of microglial phenotypes after chronic restraint stress regulated social dominancy of mice in a CX3CR1-dependent manner (unpublished data). According to the RNAseq data provided by human proteome atlas, *Grn* (Supplementary Table 1) and *Cx3cr1* (see section “Heterogeneity in microglial morphology across CNS regions”) are both expressed at relatively high levels in the limbic system, but low in the cerebellum. They are also more enriched in the corpus callosum, which is an interesting observation (see discussion below). *Hoxb8* is nearly non-existent in the brain except the pons and medulla (Supplementary Table 1). In clinical studies, two SNPs of *CX3CR1* (V249I (rs3732379) and T280M (rs3732378)) were recently found to be associated with arterial blood volume in the healthy human brains, especially around the bilateral precuneus and the left posterior cingulate and parietal cortices [119], thereby implying a region-specific role of the SNPs. GRN was suggested to be a plasma biomarker for bipolar disorder (BD) [120], but no imaging genetics data on this gene are available yet.

Microglia-driven changes in synaptic plasticity play a role in major depressive disorder as well [121]. Regional variations of microglial molecules in peripheral inflammatory conditions relevant for depression-like sickness behaviors have also been reported. For instance, an enhanced IL-1β response to peripheral *E. coli*-mediated immune challenge was only found in the hippocampus but not several other regions of the aging CNS [122]. Likewise, bacterial endotoxin lipopolysaccharide (LPS)-induced endotoxemia led to a more vigorous expression of *Il1b* in the cortex than the cerebellum, accompanied with reduced cortical expression of cholinergic genes [123]. Another paper demonstrated that when mixed neuron-glia cultures derived from the rat hippocampus, cortex, or mesencephalon were treated by LPS, mesencephalic cultures became more sensitive with a production of inflammatory factors and a loss of dopaminergic along with other neurons than hippocampal or cortical cultures, indicating that region-specific susceptibility of neurons to LPS was attributable to differences in the number of resident microglia [32].

In a poly(I:C)-induced maternal immune activation (MIA) mouse model, which phenotypically resemble schizophrenia (SCZ) and autistic spectrum disorder (ASD) [124], many cytokines in the offspring brains were found to be expressed in a region-specific manner at postnatal stages [125]. For instance, in the frontal and cingulate cortices, proinflammatory cytokines were elevated at birth, decreased during periods of synaptogenesis and plasticity, and increased again in adulthood [125]. A recent study using positron emission tomography with radiolabeled ligand selective for the 18-kDa translocator protein (TSPO), a most widely used imaging biomarker for neuroinflammation [126], found that TSPO density was decreased in the median prefrontal cortex but not the hippocampal CA or the dentate gyrus in adult offspring of MIA-treated mice [127], indicating that MIA led to long-lasting region-specific changes that may alter CNS development and behavior. However, although TSPO has been regarded as a microglial marker in clinical studies, it is abundantly expressed in not only microglia but also other brain cell types, such as astrocytes and vascular endothelial cells [127].

As discussed in previous sections, microglia in the WM represent a unique population that discerns themselves from the other regional counterparts phenotypically and functionally. WM abnormalities are commonly detected in SCZ, BD, and ASD, and contribute to cognitive deficits in these patients [128, 129]. Using diffusion tensor imaging, we recently reported significant reductions in fractional anisotropy in most regions of interest representing all major WM fasciculi, especially the anterior corona radiata and corpus callosum, in a largest-ever international cohort of SCZ patients, as compared with healthy subjects, thereby confirming a structural dysconnectivity hypothesis in SCZ [130]. SCZ involves a profound dysregulation of myelin-associated gene expression, reductions in oligodendrocyte numbers, and marked abnormalities in the ultrastructure of myelin sheaths [131]. In this sense, microglial subset along with its specific receptors would be envisaged as an active player in WM pathologies of psychiatric disorders. Corroboratively, CX3CR1 was for instance found to be significantly down-regulated in SCZ [132] and a genetic variant in CX3CR1 (A55T) that disrupted signaling of CX3CR1, which is highly expressed in the corpus callosum in mice, was associated with SCZ and ASD in humans [133]. It would be necessary to understand more deeply how this receptor as well as other candidate genes may contribute to region-specific disease development in the future.

## Conclusion and perspectives

In summary, microglia differ in their abundancy, morpho-molecular signatures, and homeostatic functions in different anatomical locations of the healthy CNS (Table 1). These heterogeneous features may lead to diversified responses of microglia toward pathological stimuli at different locations, and possibly also in time- and gender-dependent manners. As most of the clinical trials on generic immunosuppressants or anti-inflammatory drugs have failed for both neurodegenerative diseases and psychiatric disorders so far [134, 135], this strongly suggests that targeting only a subset of microglia, or other glial subtype, in a region-specific manner rather than globally, would have more promising therapeutic efficacy. But in the lack of appreciation on the diverse regional features of microglia, a global microglial inhibition seemed to have been the only solution for neuroscientists and clinicians. In this sense, understanding region-specific features of microglia, e.g., by knowing what they are, where they are from and where they are destined to, may prove utterly valuable for developing targeted modulatory therapies for treating various CNS disorders. However, gaps in our current knowledge on microglial heterogeneity prompts further investigation before it can be applied in real clinical practices. As elaborated in the above sections, there are still many aspects concerning regional heterogeneity of microglia left unaddressed. For example, it is still unclear what may be the link between molecular and morphological heterogeneity, whether there is plasticity in phenotypic and functional transformation, whether and how different microglial progenitors contribute to such regional features, and what key intrinsic program(s) or environmental cue(s) may drive such fundamental spatial differences. Hopefully, equipped with modern genetic and cell lineage tracing tools, high-throughput sorting and high-resolution sequencing technologies, as well as in vivo cell transplantation and live imaging methods, more knowledge on region-specific features will help us more deeply comprehend microglial biology in both the healthy and disordered brains.

## Supplementary information

Supplemental material

Supplementary Fig. 1

Supplementary Table 1
