# Supplementary material for: Microglial regional heterogeneity and its role in the brain
Source: Mol Psychiatry. 2019 Nov 26;25(2):351–67. doi: 10.1038/s41380-019-0609-8 (PMC6974435; doi:10.1038/s41380-019-0609-8)
Supplement: Supplementary file 1 — Supplemental material [file 41380_2019_609_MOESM1_ESM.docx]

# Supplementary method and materials

## **Flow cytometry**

10mg tissues of the cerebral cortex, thalamus, cerebellum, and spinal cord from five C57BL/6J male adult mice were dissected, immediately minced and filtered through 70µm cell strainers (#352350, BD Biosciences) in RPMI medium on ice. Homogenates were blocked with 10% rat serum (30min) and incubated with diluted fluorescent markers (all Biolegend) of MHCII-PE (#107608), CD206-FITC (#141704), F4/80-PE/Cy7 (#123114) and CD45-APC (#103112) in 0.1M PBS supplemented with 1% fetal calf serum (1h, +4°C). After washing, cell pellets were resuspended and filtered through 35μm cell strainers into flow tubes (#08-771-23, BD Biosciences). Acquisition under CD45-gating was done by a two-laser flow cytometer-Gallios (Beckman Coulter). Data were analysed using Kaluza (Beckman Coulter). Cells were quantified as % of microglia among total brain cells and MHCII^+^% or CD206^+^% among total microglia.

**Suppl. Table 1. Microglial signature gene** **RNAseq data across thirteen mouse brain regions.**

## **Suppl. Figure 1. Microglial density and polarization vary among the CNS regions in naïve C57BL/6J male mice.** Microglia from freshly isolated brain regions of 8 mice were studied by flow cytometry. (**A & B**) Cell gating strategy used to quantify total microglia among brain cells and MHCII^+^ (CD45^mid^F4/80^+^MHCII^+^CD206^-^) and CD206^+^ (CD45^mid^F4/80^+^MHCII^±^CD206^+^) microglial subpopulations. (**C**) Abundancy (%) of microglia varies in brain regions. (**D**) MHCII^+^/CD206^+^ microglial ratio, e.g. microglial polarization also varies in brain regions. Data are presented as mean ± SD; 1-w-ANOVA; * p < 0.05, ** p < 0.01, *** p < 0.001.
