# Supplementary figures and images for: Microglial regional heterogeneity and its role in the brain
Source: Mol Psychiatry. 2019 Nov 26;25(2):351–67. doi: 10.1038/s41380-019-0609-8 (PMC6974435; doi:10.1038/s41380-019-0609-8)

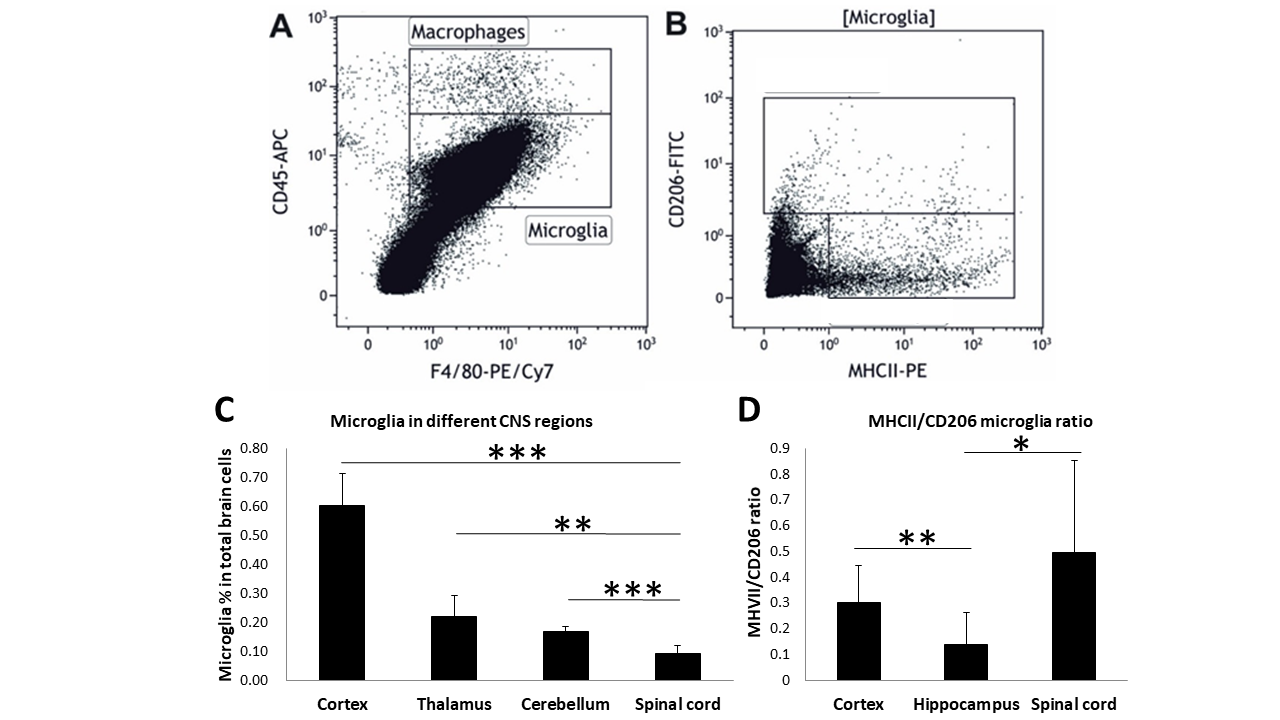

Supplement: Supplementary file 2 — Supplementary Fig. 1 [file 41380_2019_609_MOESM2_ESM.png]
